# Staying physically active during the COVID-19 pandemic: Assessing the roles of motivation, basic psychological needs, goal orientation and anticipatory sport persistence

**DOI:** 10.3389/fpsyg.2023.1057178

**Published:** 2023-03-01

**Authors:** Lotta Loerbroks, Louisa Joyce Kersten, Philipp Alexander Freund

**Affiliations:** Institute of Psychology, Faculty of Education, Leuphana University, Lüneburg, Germany

**Keywords:** self-determination theory, motivation, basic psychological needs, anticipatory sport persistence, physical activity, COVID-19, barriers

## Abstract

Nationwide barriers to public and private sport institutions were implemented during COVID-19 lockdowns. Autonomous motivation, perceived fulfillment of basic psychological needs (competence, autonomy, and relatedness) and goal orientation coincide with higher persistence rates in physical activity. The aim of this study is to investigate which factors are related to anticipatory sport persistence, a specific form of sport persistence. We conducted an online survey with *N* = 208 (74% female) participants. Correlation analyses showed that higher anticipatory sport persistence coincides with autonomous motivation (*r* = 0.314, *p* < 0.01), basic psychological needs (competence *r* = 0.528, autonomy *r* = 0.446, relatedness *r* = 0.315; all *p* < 0.01), and goal orientation (intrinsic *r* = 450, extrinsic *r* = 0.146; all *p* < 0.01). Furthermore, multiple regression analysis showed that anticipatory sport persistence can be predicted through intrinsic goal orientation (*B* = 0.465, *p* < 0.01) and the need for competence (*B* = 0.418, *p* < 0.01). The importance of anticipatory sport persistence when expecting external barriers to physical activity, its relationship toward sport persistence and possible implications for the planning and perseverance of physical activity plans are being discussed.

## 1. Introduction

Humans cope differently when encountering barriers. The outbreak of COVID-19 at the beginning of 2020 led to a global pandemic (World Health Organization, 2020). A variety of restrictions to daily life followed (European Centre for Disease Prevention and Control, 2022b).^1^ In Germany, like in most other countries, measures to confine the spread of the virus included national lockdown(s), which among other measures featured a temporary shutdown of public and private sport institutions (European Centre for Disease Prevention and Control, 2022a). This shutdown presented itself as a unique and novel barrier to exercise, as it was a governmental barrier effective nationwide, in contrast to individual or situational barriers. The barrier was effective regardless of motivation or willingness to exercise. The only exercise options were activities that could be performed individually and without interpersonal contact (European Centre for Disease Prevention and Control, 2022a). Recent studies have provided different findings concerning the amount of physical activity carried out during COVID-19 lockdowns. While some studies report a decline in exercise (Meyer et al., 2020; Stanton et al., 2020), other findings suggest an increase (Ding et al., 2020) or a mixture of both at the same time, indicating potential differences between individuals (Brand et al., 2020).

The nationwide shutdowns of public and private sport institutions were a unique phenomenon affecting the whole population (European Centre for Disease Prevention and Control, 2022a). It can be assumed that this will not be the last time that individuals will be faced with barriers to exercise options. In the current study, we take a closer look at the underlying mechanisms that might influence anticipatory sport persistence when faced with barriers. More specifically, we aim to explore the effects of motivation, goal orientation, basic psychological needs and previous physical activity on anticipatory sport persistence when faced with another hypothetical lockdown due to a pandemic situation as a unique external barrier to physical activity.

Physical activity is essential for the wellbeing of humans. Exercise facilitates the development of essential life skills such as coordination, problem-solving, social interaction, and creativity (Ryan and Deci, 2017). Regular exercise has a beneficial influence on physical and psychological wellbeing while the lack thereof is a risk factor for developing various physical (Ross et al., 2000; Williams, 2001; Higueras-Fresnillo et al., 2018) or mental (Ashdown-Franks et al., 2020) illnesses. The beneficial effects of physical activity start in childhood (Biddle et al., 2019) and can affect the entire lifespan (Wiese et al., 2018).

Following a regular exercise plan can be understood as successful goal-orientated behavior. Persistence plays an important role in reaching goals, especially when faced with barriers. Sport persistence refers to the subjectively perceived perseverance under strenuous circumstances (Brandstätter et al., 2018). Thus, it is an important prerequisite for regular physical activity. Anticipatory sport persistence can be understood as a specific form of persistence. More specific, anticipatory sport persistence is the subjectively perceived perseverance to continue physical activity when faced with barriers. Jung and Brawley (2011) explored the impact of self-regulatory efficacy on sport persistence. They asked participants to imagine a hypothetical scenario that included barriers to physical activities. Participants with higher self-regulatory efficacy showed higher sport persistence. The concept of anticipatory sport persistence is intriguing when looking at the effect of a national lockdown on physical activity. As exercising can help prevent physical and mental illnesses, it is important to understand what influences anticipatory sport persistence and how it can be encouraged.

Self-determination theory (SDT) is a widely used framework to predict and explain behavior (Ryan and Deci, 2017). SDT has a claim to universality (e.g., Deci and Ryan, 2002; Fortier et al., 2012; Guérin et al., 2012) and incorporates the concepts of motivation, basic psychological needs and goal orientation.

Within SDT, the quality of motivation refers to its source, of which several variants are assumed. These range from amotivation over forms of extrinsic motivation up to intrinsic motivation. Extrinsic motivation is differentiated into external, introjected, identified, and integrated regulation. The former two are forms of controlled motivation while the latter two, together with intrinsic motivation, are forms of autonomous motivation (Deci and Ryan, 1985, 2002). The more a motive is internalized the more autonomous motivation becomes (Guérin et al., 2012). SDT also assumes three basic psychological needs (autonomy, competence and relatedness; Ryan and Deci, 2000a; Deci and Ryan, 2002). Fulfillment of these needs shows a positive impact on health (Deci and Ryan, 2008), performance (Guérin et al., 2012), persistence and quality of motivation (Ryan and Deci, 2019) as well as psychological wellbeing (Ryan and Deci, 2000b). The process of internalizing motivation is supported by the fulfillment of the basic psychological needs (Ryan and Deci, 2019). Furthermore, according to SDT, goal orientation can be differentiated into extrinsic and intrinsic goal orientation (Ryan and Deci, 2017). Intrinsic goals correlate positively with basic psychological needs and, as a consequence, with wellbeing and persistence (Ryan and Deci, 2019). Taken together, these concepts play a vital role in explaining behavioral differences.

Self-determination theory suggests that the more autonomous a motivation is for a certain behavior, the more frequently said behavior is going to be executed. Previous research has shown a positive effect of autonomous forms of motivation on physical activity, e.g., in preventing injuries (Chan and Hagger, 2012), promoting higher self-efficacy toward physical challenges (Thøgersen-Ntoumani and Ntoumanis, 2006), perseverance in following training schedules during rehabilitation (Russell and Bray, 2010), and athletic achievement (Gillet et al., 2009). The positive association between autonomous forms of motivation and physical activity has been found to start in childhood (Owen et al., 2014). A meta-analysis by Sierra-Díaz et al. (2019) shows the positive influence of intrinsic motivation on active participation in physical exercises in students. In contrast, controlled forms of motivation can lead to higher dropout rates (Calvo et al., 2010), burnout (Jowett et al., 2013), antisocial behavior toward opponents (Hodge and Lonsdale, 2011) in athletes, and social anxiety regarding the body (Thøgersen-Ntoumani and Ntoumanis, 2006). Autonomous motivation correlates with increased sport participation and activity (Ingledew and Markland, 2008), whereas controlled forms of motivation and amotivation can have a negative impact on physical activity (Craike, 2008). Intrinsic and identified motivation have been shown to correlate positively with persistence (Deci and Ryan, 2000) and frequency (Teixeira et al., 2012) of physical activity. Furthermore, autonomous forms of motivation are predictors for long-term sport persistence (Pelletier et al., 2001). Overall, previous research suggests that autonomous forms of motivation are strong predictors for sport persistence, whereas external regulation negatively impacts sport persistence.

The perceived fulfillment of basic psychological needs also has a positive impact on physical activity. Exercise regimes that support autonomy of athletes correlate with the perceived fulfillment of autonomy and competence as well as with autonomous motivation (Isoard-Gautheur et al., 2012). The fulfillment of basic psychological needs before the start of a competitive game coincides with better performances by athletes, and good performance can lead to enhancement in competence and relatedness (Sheldon et al., 2013). Athletes that have control over their behavior (autonomy), experience success (competence) and feel valued (relatedness) are more actively engaged and persistent in their physical activities and enjoy better physical and psychological health (Ntoumanis and Mallet, 2014), while the lack thereof can lead to negative emotional states and burnout (Bartholomew et al., 2011).

Applying SDT to physical activity shows that intrinsic goal orientation leads to better performance and sport persistence when learning a new sport (Vansteenkiste et al., 2004). Intrinsic goals facilitate fulfillment of psychological needs, better self-esteem, psychological health, and self-reported athletic behavior (Sebire et al., 2009). A study by Sheldon et al. (2004) also showed a positive correlation between intrinsic goals and autonomous motivation. Increasing the importance of intrinsic goals can be accompanied by higher fulfillment of basic psychological needs, overall wellbeing and higher persistence rates (Ryan and Deci, 2019).

When looking at the effectiveness of SDT on interventions toward health behavior changes or the increase of physical activity, recent meta-analyses show a small mediating effect for SDT-based interventions (Sheeran et al., 2020; Rhodes et al., 2021). Interventions that increase autonomous motivation and perceived competence can lead to health behavior changes (Sheeran et al., 2021).

Taken together, these findings show that SDT can be used to explain differences in sport persistence through the aspects of motivation (e.g., Sierra-Díaz et al., 2019), perceived fulfillment of basic psychological needs (e.g., Ntoumanis and Mallet, 2014) and goal orientation (e.g., Vansteenkiste et al., 2004) or a combination of these aspects (e.g., Sebire et al., 2009). These findings are concerned with sport persistence, but little is known about the influence of these aspects on anticipatory sport persistence. Seeing that there is a possibility of new individual, institutional or governmental barriers to physical activity in the case of another potential future pandemic, we wonder if anticipatory sport persistence, as an important precursor of actual sport persistence, is also connected to the principal motivational elements of SDT. In this study we aim to better understand the influences of motivation, goal orientation and perceived basic psychological needs on anticipatory sport persistence when faced with a hypothetical lockdown due to a pandemic (such as COVID-19). Based on this, we assume the following hypotheses:

*Hypothesis* 1: The quality of motivation correlates positively with anticipatory sport persistence.

*Hypothesis* 2: Each of the perceived fulfillment of each of the basic psychological needs (autonomy, competence and relatedness) in chosen physical activity correlates positively with anticipatory sport persistence.

*Hypothesis* 3: Intrinsic and extrinsic goal orientation correlate positively with anticipatory sport persistence, with the magnitude of the relationship being larger for intrinsic goal orientation.

*Hypothesis* 4: Anticipatory sport persistence can be predicted jointly by (1) quality of motivation, (2) perceived fulfillment of basic psychological needs in a chosen physical activity, and (3) goal orientation.

## 2. Methods

### 2.1. Statistical analysis

Hypotheses 1–3 were tested using bivariate correlation, whereas Hypothesis 4 was tested using multiple regression analysis. All analyses were performed with the software JASP (JASP Team, 2022).

We conducted an *a priori* power analysis (Maxwell et al., 2008) with G*Power (Faul et al., 2007, 2009). For a regression model assuming a medium-sized effect of *f*^2^ = 0.15, a significance level of *α* = 0.05 and *k* = 6 predictor variables, a sample size of *N* = 146 was calculated as possessing a statistical power of 1–*β* = 0.95.

### 2.2. Sample and procedure

Recruitment took place in the second half of the calendar year 2021. Participants were recruited through an internal university recruitment system, social media, flyers in local athletic and outdoor shops as well as in local fitness centers. Data were collected anonymously via an online survey questionnaire, generated and administered using SoSci-Survey (Leiner, 2019). Participation was voluntary and anonymous. At the end of the questionnaire participants had the option to join in a raffle for three 10€-vouchers, and psychology students from Leuphana University received credit points for participation. Contact data for the raffle and credit points were collected separately. The final sample consisted of *N* = 208 participants (74% female) with a mean age of *M* = 26.76 years (*SD* = 11.45). Further sociodemographic data are detailed in Table 1.

**Table 1:** Sociodemographic characteristics of participants.

	*n*	Percentage
Gender		
Female	154	74
Male	54	36
Highest completed education level		
Attending school	8	3.8
Middle school or lower	10	4.8
High school diploma	145	69.7
Bachelor or master degree	42	20.2
Other educational level	4	1.9
Occupation		
Student (school level)	7	3.4
Apprenticeship	8	1.4
Student (university level)	144	69.2
Salaried employee	50	24
Other/unspecified	3	1.4

*N* = 208. Participants were on average 26.76 years old (*SD* = 11.45).

### 2.3. Measurements

#### 2.3.1. Sport motivation

Sport motivation was measured with the Sport Motivation Scale II (SMS-II) developed by Pelletier et al. (2013). The original items were translated into German by the third author of the present study and then translated back into English by a native English speaker. All discrepancies in meaning were then resolved through discussion. The SMS-II comprises seven factors of sport motivation ranging from amotivation to intrinsic motivation, based on SDT. For the present study, we used the subscales intrinsic motivation (*α* = 0.735), identified motivation (*α* = 0.815), introjected motivation (*α* = 0.702), and external motivation (*α* = 0.648), each of which were assessed with three items and using a 7-point rating scale (1 = *does not correspond at all* to 7 = *corresponds completely*). Based on these four subscales, we computed the so-called Relative Autonomy Index (RAI; *cf.*, Howard et al., 2020) for each participant^2^. A high RAI score reflects a strong degree of autonomous motivation, whereas a low score indicates a rather strong reliance on controlled motivation. Table 2 presents descriptive statistics (*M* and *SD*) for the RAI.

**Table 2:** Descriptive statistics and correlations for study variables.

	*M* (*SD*)	RAI	Aut	Comp	Rel	InG	ExG
RAI	4.957 (3.716)						
Aut	5.136 (1.083)	0.392^**^					
Comp	5.059 (1.147)	0.350^**^	0.713^**^				
Rel	4.653 (1.331)	0.343^**^	0.487^**^	0.524^**^			
InG	5.317 (0.805)	0.408^**^	0.427^**^	0.489^**^	0.428^**^		
ExG	4.663 (1.262)	−0.075	0.109	0.163^**^	−0.016	0.528^**^	
AS	5.365 (1.390)	0.314^**^	0.446^**^	0.528^**^	0.315^**^	0.450^**^	0.146^*^

*N* = 208; RAI = relative autonomy index; Aut = autonomy; Comp = competence; Rel = relatedness; InG = intrinsic goals; ExG = extrinsic goals; AS = anticipatory sport persistence. ^*^p < 0.05; ^**^p < 0.01.

#### 2.3.2. Basic psychological needs

Basic psychological needs were assessed with the German version of the *Psychological Need Satisfaction Questionnaire* (PNSE; Wilson et al., 2006) by Rackow et al. (2013), consisting of the subscales autonomy, competence, and relatedness. Items were rated on a 7-point rating scale ranging from 1 (*I do not agree at all*) to 7 (*I agree very strongly*). For autonomy, we yielded a reliability estimate of *α* = 0.579, for competence, *α* = 0.819, and for relatedness, *α* = 0.806. Descriptives for these three subscales are presented in Table 2.

#### 2.3.3. Goal orientation

Exercise based goal orientation was assessed with the German version of the *Goal Content Exercise Questionnaire* (GCEQ; Sebire et al., 2008) by Kleinert et al. (2010). The GCEQ features 20 items covering the five dimensions social affiliation, health management, skill development, image, and social recognition. Each dimension was assessed with four items each using a 7-point rating scale ranging from 1 (*not at all important*) to 7 (*extremely important*). Social affiliation (*α* = 0.797), health management (*α* = 0.678) and skill development (*α* = 0.811) were combined into a score representing intrinsic goals (*α* = 0.800), and the combination of image (*α* = 0.935) and social recognition (*α* = 0.853) constituted an extrinsic goals score (*α* = 0.887). Descriptive statistics for intrinsic and extrinsic goal orientation are presented in Table 2.

#### 2.3.4. Anticipatory sport persistence

In order to measure anticipatory sport persistence, a mental scenario was introduced that included a barrier in order to activate persistence. Participants were asked to imagine another lockdown starting the next day that would lead to closure of public and private sport institutions and the prohibition of team and contact sports. All items on anticipatory sport persistence were to be answered with this scenario mentally in effect. Anticipatory sport persistence was measured with a German version of the anticipatory sport persistence scale by Jung and Brawley (2011). The original items were adapted to German by the second author of the present paper employing the back-and-forth translation method. Four items assess time, effort, persistence and attention toward the continuation of athletic activities during the imagined lockdown scenario on a 7-point rating scale ranging from 1 (*not at all*) to 7 (*as much as is needed to continue exercises*). For this index score, *α* = 0.929, and descriptives are listed in Table 2.

## 3. Results

Table 2 features all correlations between the scales used in the present study. The correlation between anticipatory sport persistence and the RAI-score as a reflection of an individual’s most typical motivational source of behavior regulation was *r* = 0.314, *p* < 0.01, illustrating a positive relationship of medium size according to the standards suggested by Cohen (1988). Autonomous motivation was connected with higher anticipatory sport persistence. Thus, Hypothesis 1 was supported by the empirical data.

All three basic needs correlated positively with anticipatory sport persistence as well (competence: *r* = 0.528, autonomy: *r* = 0.446 and relatedness: *r* = 0.315; all *p* < 0.01). Only the difference in the magnitude of the correlations between competence and relatedness was significant (*z* = 2.65, *p* < 0.01), whereas there was no statistically significant difference between the correlations for competence and autonomy or between autonomy and relatedness. Overall, these results support Hypothesis 2.

Hypothesis 3 could also be supported. While both relationships were positive and significant, the correlation with anticipatory sport persistence was significantly larger for intrinsic goal orientation than for extrinsic goal orientation (*z* = 3.42, *p* < 0.01).

We performed a multiple regression analysis in order to test Hypothesis 4. The resulting multiple correlation was estimated as *R* = 0.582 (*R*^2^ = 0.339, *R*^2^_adj_ = 0.319). Table 3 details the results of the regression model. Of the six predictors, only intrinsic goal orientation and the need for competence reached statistical significance, rendering the remaining variables unnecessary for the prediction of anticipatory sport persistence. In total, the results of this analysis support Hypothesis 4.

**Table 3:** Results of regression analysis predicting anticipatory sport persistence.

*Predictor*	*B*	*SE*	*Beta*	*t*	*p*
(Intercept)	0.599	0.584			
RAI	0.022	0.026	0.059	0.847	0.398
Autonomy	0.115	0.109	0.090	1.059	0.291
Competence	0.418	0.106	0.345	3.927	< 0.01
Relatedness	−0.048	0.076	−0.046	−0.623	0.534
Intrinsic goals	0.465	0.155	0.269	3.008	< 0.01
Extrinsic goals	−0.065	0.083	−0.059	−0.781	0.435

*N* = 208. *B* = unstandardized regression coefficient; *SE* = standard error; *Beta* = standardized regression coefficient; *RAI* = relative autonomy index.

## 4. Discussion

Based on SDT (Ryan and Deci, 2017), the present study analyzed the associations between motivation, perceived psychological needs and goal orientation with anticipatory sport persistence under a hypothetical pandemic-induced lockdown. Moreover, we sought to examine their respective relative influence for the prediction of anticipatory sport persistence. The main findings were that all variables of interest were significantly associated with anticipatory sport persistence. Furthermore, we found that anticipatory sport persistence can be predicted significantly through intrinsic goal orientation and the need for competence.

In support with Hypothesis 1, autonomous motivation was associated with higher anticipatory sport persistence under a hypothetical lockdown. Previous studies showed that autonomous motivation is associated with sport participation (Ingledew and Markland, 2008), persistence (Deci and Ryan, 2000) and frequency of physical activity (Teixeira et al., 2012). Together with our findings, this suggests that anticipatory sport persistence might not only be associated with motivation itself but also with decisive factors for the planning of physical activities when faced with external barriers. Fostering autonomous motivation might be a key factor in the planning process for physical activity in order to strengthen anticipatory sport persistence, especially when facing external barriers. It would be interesting to explore if the same effect is found when faced with individual or institutional barriers, as our study focused on barriers through a pandemic (such as COVID-19)-induced lockdown.

The present study demonstrated that the fulfillment of basic psychological needs is associated with anticipatory sport persistence, supporting Hypothesis 2. More specifically, the need for competence showed the strongest association with anticipatory sport persistence, followed by autonomy and relatedness. Previous studies examined the association between autonomy and motivation (Isoard-Gautheur et al., 2012), fulfillment of basic psychological needs before and during physical activity (Sheldon et al., 2004) and their associations with persistence (Ntoumanis and Mallet, 2014). Our findings contribute to these findings by showing the importance of fulfilling basic psychological needs not only before or during physical activity, but also when anticipating physical activity while facing barriers. This could have implications for planning and anticipating physical activity even when expecting barriers. Creating contexts that support individual’s competence, autonomy and relatedness could support the development of higher anticipatory sport persistence and thus lead to higher physical activity.

With Hypothesis 3, we assumed that intrinsic and extrinsic goal orientation are associated with anticipatory sport persistence. In accordance with our hypothesis, we found that both forms of goal orientation are associated with anticipatory sport persistence, with a significantly higher effect for intrinsic goal orientation. This shows that intrinsic goal orientation is not only relevant during physical activity (Vansteenkiste et al., 2004), but already comes into effect when individuals are thinking about upcoming physical activity in the form of anticipatory sport persistence.

With Hypothesis 4, we assumed that anticipatory sport persistence can be predicted through quality of motivation, perceived fulfillment of basic psychological needs in a chosen physical activity, and goal orientation. Contrary to our hypothesis, only intrinsic goal orientation and the need for competence as one of the basic psychological needs were predictors for anticipatory sport persistence. Although we found individual associations between these factors and anticipatory sport persistence, not all of them were predictors. Therefore, strengthening intrinsic goal orientation and the need for competence could lead to higher anticipatory sport persistence. The relationship between the other factors and anticipatory sport persistence should be further explored, in order to better understand the individual associations and predictive factors.

Some further points have to be taken into consideration when discussing our results. The present study was conducted after the first COVID-19 induced lockdown in Germany. Participants experienced the impact of a lockdown shortly before data collection and knew from experience how such a barrier could impede their actual engagement in physical activity. Their reported anticipatory sport persistence might have been influenced by additional factors such as the psychological stress of the lockdown (Meyer et al., 2020; Stanton et al., 2020), a general uncertainty at that time and further individual factors, such as childcare or working remotely from home. These factors were not explored in more detail at the time of data collection. Research about the complexity of individual situations during the COVID-19 lockdowns is still ongoing. We suggest to further explore the relationship between anticipatory sport persistence and different forms of barriers.

Based on our findings, we can conclude that SDT is not only a useful framework to predict and explain behavior (Ryan and Deci, 2017), but can also be applied to explain *anticipated* behavior while facing external barriers. Overall, our findings show that anticipatory sport persistence plays a role not only when imagining barriers but might also be relevant when actual barriers occur. Anticipatory sport persistence could even be a prerequisite for general sport participation. These findings might have implications for interventions to increase sport participation and to help individuals to persevere with their exercise schedule regardless of barriers and impairments.

There are some limitations to the present study that should be acknowledged. Firstly, the modest reliability for autonomy limits to what extent the relationship between autonomy and anticipatory sport persistence can be generalized. Secondly, using a convenience sample, including an uneven distribution of gender, might limit the extent to which the present findings can be applied to the population. Thirdly, the sample is relatively small, thus prohibiting the use of latent variable modeling for data analysis. Fourthly, the data was based on self-reports. It cannot be ruled out that our results are biased by social desirability.

Despite the limitations, our findings provide valuable insight into understanding anticipatory sport persistence and even bring about implications for supporting individuals in maintaining physical activity when faced with barriers. We suggest an exploration of the relationship between anticipatory sport persistence before facing barriers with sport persistence while facing barriers as this might help to better understand the inconsistent findings concerning the amount of physical activity carried out during the first lockdowns (Brand et al., 2020; Ding et al., 2020; Meyer et al., 2020; Stanton et al., 2020). Our study included the scenario of a hypothetical anticipated lockdown as an external barrier. To better understand how anticipatory sport persistence relates to different types of barriers, it would be interesting to also include personal and institutional barriers.

Seeing that physical activity is important for physical and mental health (Ross et al., 2000; Williams, 2001; Higueras-Fresnillo et al., 2018), contributing factors for the engagement in physical activity should be nurtured. Activating anticipatory sport persistence through the promotion of intrinsic goal orientation and competence could be an important step when planning for physical activity. Interventions that promote a healthier lifestyle might be conducted before engaging in physical activity. Supporting individuals in developing higher anticipatory sport persistence could be beneficial for emerging exercise plans. Future studies should look into the relationship between anticipatory sport persistence and actual physical activity in order to further determine contributing and hindering factors for a healthier lifestyle when faced with barriers.

In conclusion, our findings suggest that anticipatory sport persistence, while faced with an imaginary lockdown due to a pandemic such as COVID-19, can be predicted through intrinsic goal motivation and the need for competence. Anticipatory sport persistence is a relatively novel research subject. Our findings contribute to the understanding of this construct and encourage further research in order to better understand implications for physical and mental wellbeing.

## Data availability statement

The raw data supporting the conclusions of this article will be made available by the authors, without undue reservation. Inquiries can be directed to the corresponding author.

## Ethics statement

Ethical review and approval was not required for the study on human participants in accordance with the local legislation and institutional requirements. The patients/participants provided their written informed consent to participate in this study.

## Author contributions

PF, LK, and LL contributed to conception and design of the study. LK organized and conducted the data collection. PF performed the statistical analyses and wrote sections of the manuscript. LL wrote the first draft of the manuscript. All authors contributed to the article and approved the submitted version.

## Funding

We acknowledge support by the German Research Foundation (DFG) and the Open Access Publication Fund of Leuphana University Lüneburg.

## Conflict of interest

The authors declare that the research was conducted in the absence of any commercial or financial relationships that could be construed as a potential conflict of interest.

## Publisher’s note

All claims expressed in this article are solely those of the authors and do not necessarily represent those of their affiliated organizations, or those of the publisher, the editors and the reviewers. Any product that may be evaluated in this article, or claim that may be made by its manufacturer, is not guaranteed or endorsed by the publisher.

## Footnotes

^1^Due to the dynamic development of infection rates and measurements against COVID-19, we refer to the European Centre for Disease Prevention and Control (2022b, 2023)  and the World Health Organisation (2023) who frequently update new findings, (effective January 2023).

^2^RAI computed as: (−2*External) + (−1*Introjected) + (1*Identified) + (2*Intrinsic).
